# Sense of Smell as the Central Driver of Pavlovian Appetite Behavior in Mammals

**DOI:** 10.3389/fphys.2019.01151

**Published:** 2019-09-18

**Authors:** Leon G. Fine, Celine E. Riera

**Affiliations:** ^1^Department of Biomedical Sciences, Cedars-Sinai Medical Center, Los Angeles, CA, United States; ^2^Program in the History of Medicine, Cedars-Sinai Medical Center, Los Angeles, CA, United States; ^3^Center for Neural Science and Medicine, Cedars-Sinai Medical Center, Los Angeles, CA, United States; ^4^Board of Governors Regenerative Medicine Institute, Cedars-Sinai Medical Center, Los Angeles, CA, United States

**Keywords:** appetite and energy expenditure, food perception, palatability, hypothalamus, ghrelin

## Abstract

The seminal experiments of Ivan Petrovich Pavlov set the stage for an understanding of the physiological concomitants of appetite and feeding behavior. His findings, from careful and creative experimentation, have been uncontested for over a century. One of Pavlov’s most fundamental observations was that activation of salivary, gastric and pancreatic secretions during feeding and sham-feeding, precedes entry of food into the mouth, generating signals to the brain from various sensory pathways. Pavlov referred to this as the “psychic” phase of digestion. However, quite surprisingly, he did not attempt to isolate any single sensory system as the main driver of this phenomenon. Herein we revisit Pavlov’s findings and hypothesize that the evolutionarily-important sense of smell is the pathway most-likely determinant of feeding behavior in mammals. Substantial understandings of olfactory receptors and their neural pathways in the central nervous system have emerged over the past decade. Neurogenic signals, working in concert with hormonal inputs are described, illustrating the ways in which sense of smell determines food-seeking and food-preference. Additionally, we describe how sense of smell affects metabolic pathways relevant to energy metabolism, hunger and satiety as well as a broad range of human behaviors, thereby reinforcing its central biological role in mammals. Intriguing possibilities for future research, based upon this hypothesis, are raised.

## Introduction

In the context of food, appetite surely means “a desire for eating.” Expressed in a more exaggerated way, it could be “a passionate longing for food.” Both descriptive phrases were used by Ivan Petrovitch Pavlov (1849–1936) in his “Lectures on the work of the digestive glands” published in 1897, which was translated into English by Pavlov and Thompson (1902) and re-issued in Russian and English in Pavlov and Thompson (1982).

We examine herein the seminal discoveries of Pavlov related to appetite and feeding and explore new interpretations of some of his findings in the light of recent insights into the science of olfaction. We argue that there was a void in his exploration of the phenomenon of appetite, with specific regard to the sense of smell, and that this void was not filled by experimentation for over a century. We hypothesize that sense of smell is a central driver of appetite, food-seeking, food preference in vertebrates, including humans. We further argue that, if sense of smell is indeed central to these functions, from an evolutionary standpoint, it is likely that it also participates in other, wider physiological functions. We provide evidence from the literature for the existence of a number of pathways via which smell influences behavior and metabolism.

## The Experiments and Conclusion of Pavlov

### Pavlov the Physiologist

Pavlov, a Nobel Prize-winning physiologist, was born in Russia and studied at the St Petersburg University and the Medico-chirurgical Academy, from which he graduated in 1879. Thereafter, he worked as the laboratory head at the Military Medical Academy in St. Petersburg, before moving abroad in 1884 to study with Ludwig in Leipzig and Heidenhain in Breslau, both being physiologists of the highest repute at the time. He returned to the Military Academy in 1888, where he discovered the secretory nerves to the pancreas. In the following year, he began his experiments on sham-feeding.

He ultimately obtained a chair in pharmacology and in 1891 was appointed as Director of the Department of Physiology in the Institute of Experimental Medicine. He became renowned for his recognition of the conditioned reflex. Pavlov’s work focussed mainly on the neural control of organ function, an interest which was sustained despite the emergence of contemporaneous discoveries of humoral control of gastrointestinal function at the time, including the discovery of secretin, a stimulant of pancreatic secretion, by Starling (Fine, 2014).

Pavlov’s skill as a surgeon was legion, as was his obsessive attention to experimental detail, his use of antiseptic techniques and his imaginative surgically created experimental models. The conclusions he drew from his experiments never exceeded the realities of the data generated.

Below we review Pavlov’s discovery of the stimulatory effects of appetite on the initiation of gastrointestinal secretion and his neuro-physiological explanations for such. We then focus on current discoveries related to the sense of smell and olfaction as a driver of food-seeking, appetite-stimulation and taste perception, to which Pavlov payed specific attention.

### Pavlov Revisited: End-Organ Response Patterns

In his lectures Pavlov posed a series of pointed questions about gastrointestinal end-organ function, which provided the direction for his studies (Pavlov and Thompson, 1982). These related to the quantities and constituents of secreted fluids in different parts of the gastrointestinal tract, their inter-relationships and their variations in response to the food ingested. Here we focus on appetite, which precedes the phase of food ingestion and which drives the search for food, the preferences for certain foods and the underlying physiological processes which govern the phenomenon of appetite. The details are of importance since they reinforce the care and creativity which Pavlov brought to his work.

Pavlov proclaimed: “I hope to furnish you with evidence sufficiently convincing, that the alimentary canal is endowed, not with mere general excitability, (that is to say, does not respond to every conceivable form of agency), but only to special conditions which are different for different portions of its length.”

He continues: “Gastric and pancreatic glands have, what we may call, an instinct. They pour out their juice in a manner which exactly corresponds, both qualitatively and quantitatively to the amount and kind of food partaken of. Moreover, they secrete precisely that quality of fluid which is most advantageous for the digestion of the meal. We naturally ask ourselves at once, by what means is this made possible? On what does this instinct of the glands depend, and in what does it consist? A probable answer to this question is easily given and, naturally, an explanation of the adaptability of the glands is, above all, to be sought for in their innervation”(Pavlov and Thompson, 1982).

It had been shown previously, in experiments on dogs and later in a patient with a surgically created gastrotomy, that gastric secretion was induced prior to any contact of food with the stomach. Pavlov thus needed to set up experiments which would allow him to determine the secretory responses to raw materials to be found in separate portions of the digestive canal and which would identify the stimuli to such responses.

### Pavlov’s Experimental Models

**FIGURE 1:**
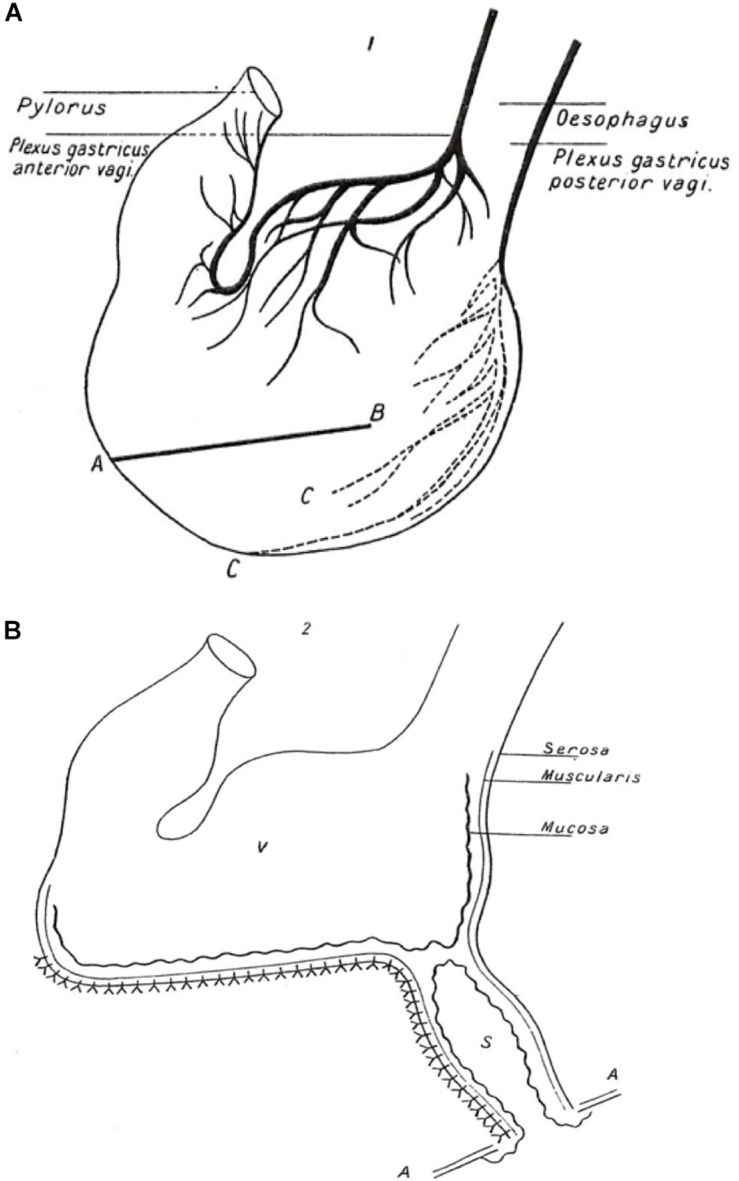
Illustration of Pavlov’s gastric pouch. A copy of the illustration published in the 1902 English translation of Pavlov’s “Work of the digestive glands” (Pavlov and Thompson, 1902, 1982). (The figure was originally created by Pavlov for the 1897 publication in Russian, the wording having been converted into English). **(A)** Anatomy of stomach showing anterior and posterior plexuses of the vagus nerve. AB shows line of incision through anterior and posterior walls of the stomach to form a triangular flap (not shown) in area C. An incision was made at the base of the flap only through the mucous membrane, the muscular and peritoneal coats remaining intact. Out of a piece of the flap a cupola was formed (not shown) to create an innervated pouch separate from the main body of the stomach. **(B)** Gastric pouch (S) with intact mucous membrane opening onto the surface of the abdominal wall (AA) and separated from the main body of the innervated stomach (V) Nerve endings are not shown. Sampling of gastric fluid was performed through the open end of the pouch.

Pavlov’s experimental animal was the dog. He needed to be able to measure salivary, gastric and pancreatic secretion quantitatively as a function of time. Being a highly skilled surgeon, he was able to exteriorize the salivary ducts and the pancreatic duct and to create an isolated gastric pouch (Figure 1) which retained it innervation but was separated from the main body of the stomach, so that its secretions could be sampled via an exteriorized opening in the absence of contact with food. By observing the time courses of secretion of these individual organs he could determine whether or not they acted in sequence and whether they responded to similar signals.

An important experimental model was that of “sham feeding,” in which the esophagus was sectioned, the proximal portion being exteriorized and the distal portion closed off. Although the animal could eat hungrily, all the food swallowed would exit the body via the esophageal stoma. Dogs could be fed this way for hours, since satiety was not induced, and secretion of gastric juice (sampled from the blind pouch) continued for the duration of the experiment.

### The “Psychic Phase” and Physical-Chemical Stimulation of the Salivary and Gastric Glands

The phrase “to make one’s mouth water” refers figuratively to the phenomenon of salivation prior to putting food into one’s mouth. Pavlov referred to saliva as the “host to every substance taken in, preparing the food for further passage if the mouth deems it acceptable for ingestion.” He saw this as a specific physiological “sense” independent of the stimulation of buccal nerve endings.

Regardless of what foodstuffs Pavlov tested, the salivary and gastric responses to food placed into the mouth of his experimental animals, without it entering the stomach (sham feeding), elicited little or no immediate secretory responses. On the other hand, tempting the dogs with food for only 5 min, did induce secretion. The more eager the dogs were to eat, the greater was the volume of the secretions. He further observed that dogs demonstrated food preferences which he ascribed to the sense organs collectively. For instance, in contrast to that observed with meat or bread, his dogs showed little interest in milk and very little gastric secretion accompanied its ingestion. On the other hand, the parotid response to introduction of flesh into the mouth was absent, but when powdered dry flesh was introduced an abundant secretion followed. This occurred despite the fact that the desire to eat was clearly stronger for flesh than for dry food as assessed by salivation prior to eating.

From an experimental standpoint, Pavlov says: “It is only necessary that some food be near the dog or that the hands of the attendant who has prepared the food should smell of it or that some other similar circumstance should come into play, which would incorrectly attribute gastric secretion to a physical stimulus.” However, he did not conduct experiments to isolate sense of smell as a driver of this response (Pavlov and Thompson, 1982).

Quoting Pavlov: “To restore appetite to a man means to secure him a large stock of gastric juice wherewith to begin digestion of the meal.” The act of feeding, stimulated by appetite, is transmitted to the stomach by nervous channels to the gastric glands and does not require physical contact with food. Pavlov referred to this as “psychic” stimulation. He made no attempt to separate the various sensory stimuli which constituted the pre-ingestion phase, i.e., sight, sound, smell, tactile senses, to which he referred only collectively.

### Pavlov on the Enjoyment of Eating

The satisfaction derived from eating is a combination of anticipatory preparation of secretion and tactile and taste sensation in the mouth, followed by “impulses awakened by the passage of food along deeper portions of the esophagus and by entry into the stomach.” The craving for food and the impulse to seek it, is thus the first and strongest exciter of several gastrointestinal organs. Processing of food serially along the alimentary tract, prepares each subsequent section to “harmonize” with that which it receives.

Given the primacy of expression of the pre-ingestion phase of eating, “perhaps the old and empirical requirement that food should be eaten with interest and enjoyment, is the most imperatively emphasized and strengthened of all” says Pavlov. Regarding a meal, he talks of the time of day, the company present, the special features of the room and the lack of urgency, as a means to “take away the thoughts from the cares of daily life and to concentrate on the repast.” He alludes to alcoholic beverages in helping this distraction. It is not obvious to him why bitters are used as therapeutic secretagogues, but he suggests that they perhaps stimulate appetite by first eliciting an unpleasant impulse which “awakens the idea of a pleasant one.” Meat extract and acidic foods he sees as the most effective agents to stimulate appetite.

Awakening of curiosity and interest, augments the desire for food. Quoting Pavlov: “It is only necessary to give an impulse to the organs of taste, that is, to excite them, in order that their activity may be later maintained by less powerful excitants. Even a hungry dog will not eat everything with equal pleasure but will seek out the food which it relishes best.”

## General Considerations of Feeding Behavior, Metabolism and the Neurobiology of Energy Balance

### Beyond Pavlov: Physiology of Food Reward

Where has Pavlov left us? Over a century has passed since he published his findings. He made no obvious attempt to tease out which of the senses to which he referred collectively (sight, smell, hearing, touch), accounted for the appetitive behavior patterns which he observed. The following sections address the neurohumoral pathways which govern appetite and food seeking and the central role of sense of smell in this context, arguing that this sense can probably best account for the rapid response which Pavlov described.

We have entered the era of exploration of the concept of “food reward” and its physiological underpinnings. Berridge, in his classical paper (Berridge, 1996), distinguishes between “wanting” and “’liking” food. “Wanting” corresponds to appetite or craving, whereas liking refers to the concept of palatability and the hedonic consequence of eating pleasurable food. Pavlov’s dogs “wanted” food when they had been deprived of it for some time and generated the appropriate secretory responses, which prepared them to receive it. On the other hand, when offered some foods for which they had no “liking” (milk was one), they ignored it.

Berridge contends that fundamental brain processes can be separated from the subjective experience (Berridge, 1996). His premise is that palatability, the hedonic component of food reward, results from an integrative process which melds taste with the underlying physiological and psychological states and with individual history and memory. The pleasantness of taste of a particular food varies according to the physiological state, accounting for aversion to food in a number of disease states. He contends that food reward is largely an incentive process conditioned by its own control system.

Olfactory memory and prior experience with the food, is one of the factors which act as incentives to eating, the others being the nature of the food and the state of energy repleteness or depletion (Spence and Youssef, 2015; Sullivan et al., 2015). These entities activate neural and humoral pathways which govern behavioral patterns and are conditioned by the pleasure induced by the reward. This, in turn, triggers further neural activity.

The pleasure associated with food or its palatability, in which olfaction is important, is a critical aspect of food selection and food ingestion, and which also drives meal size decisions (Harris et al., 2008; McCrickerd and Forde, 2016). Foods with high positive valence can motivate ingestion without the requirement of hunger signals (Berthoud et al., 2017). Opposing homeostatic feeding (food consumption to reload energy sources), this type of eating behavior is considered non-homeostatic. Certain brain circuits process information related to food reward, and are sensitive to the hedonic value of palatable food, thereby influencing motivation behaviors to acquire these foods. These neurons respond to metabolic and hormonal signals released within the body to inform the brain about current available energy supplies. These reward circuits have been identified in rodent studies as mesolimbic dopaminergic neurons in the ventral tegmental area (VTA), which project to the nucleus accumbens (NAc) and other forebrain areas (Kelley et al., 2005; Morton et al., 2014). Multiple hypothalamic neurons send projections into these reward centers, such as a hypothalamic nucleus termed the lateral hypothalamic area (LHA) and proposed to integrate reward-related input with information related to energy homeostasis (Leinninger et al., 2009). Functional magnetic resonance imaging in humans have provided confirmation that the human hypothalamus is sensitive to the satiation state of the individual (Page et al., 2013; Luo et al., 2017).

### Current Views on Feeding and the Neurobiology of Energy Balance

**FIGURE 2:**
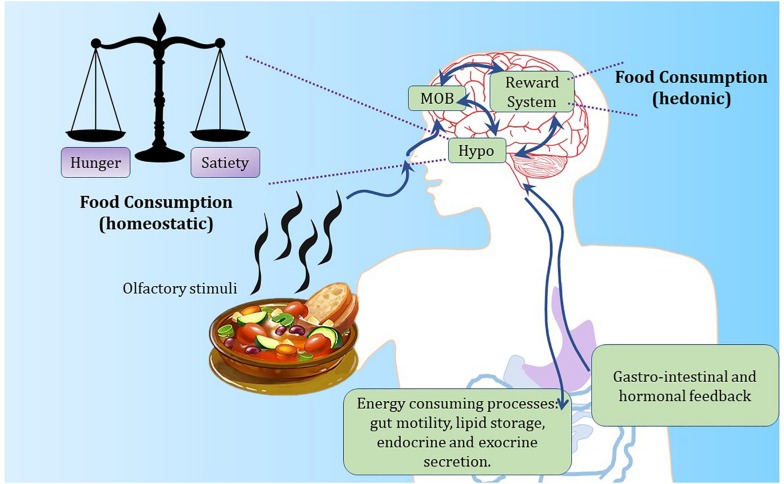
The hypothalamus regulates energy balance by integrating internal and external feeding signals. Food-based olfactory cues reach the olfactory epithelium in the nose to stimulate olfactory sensory neurons. Such neurons signal to olfactory processing centers in the brain. Olfactory cues are likely to promote hunger signals within the brain through the activation of olfactory processing neurons and hypothalamic stimulation of AgRP neurons controlling appetite. Conversely, after meal ingestion, satiety is driven by gastro-intestinal inputs and central stimulation of POMC neurons. Reward circuits play an important role in food ingestion, by influencing both hypothalamic and olfactory activity. MOB, main olfactory bulb; Hypo, hypothalamus.

Research efforts over the past 60 years in rodent models have considerably extended our knowledge of what goes on physiologically and at a molecular level before and after the ingestion of a meal, such as decisions to eat and how energy levels are maintained between meals. Energy balance is the difference between energy intake (calories eaten) and energy expenditure (calories utilized by the body). For humans and other mammals, this process is regulated by hormonal and neuroendocrine cross-talk between the brain and peripheral tissues (Figure 2). Weight gain occurs when energy intake surpasses energy expenditure, whereas weight loss is a result of energy expenditure exceeding food intake. Such an intricate balance depends on the coordinated chemical communication between vital organs and brain regions such as the hypothalamus (Schneeberger et al., 2014).

The major hormonal players in these circuits include insulin, leptin, ghrelin, and multiple gut peptides (Besser and Mortimer, 1974; Schneeberger et al., 2014). These circulating hormones secreted by peripheral tissues inform the brain of available energy stores and as a results, corrective tunings to food intake are initiated in the brain. A powerful suppressor of appetite is the adipose hormone leptin, discovered by Jeffrey Friedman’s group in 1994 (Zhang et al., 1994). Acute leptin injections powerfully suppress food intake and promote weight loss in rodent studies. However, leptin resistance observed in obese individuals potentially disqualifies leptin therapies as a cure to the obesity and type 2 diabetes epidemic (Coppari and Bjørbæk, 2012; Neill, 2013). In addition, other hormonal signals also moderately influence satiety circuits, notably the pancreatic hormone insulin, in addition to its well established role of promoting glucose uptake in peripheral tissues.

Interestingly, meal initiation is influenced by many external factors such as sensory perception of food, whereas meal size mostly depends on the release of gut peptides, notably peptide YY3–36 (PYY3–36), glucagon-like peptide 1 (GLP-1) and cholecystokinin (CCK) (Druce et al., 2004). Feeding as observed first by Pavlov is stimulated by gastric juices arising from the stomach, which have now been attributed by the hormone ghrelin discovered in Kojima et al. (1999). Ghrelin is secreted before meal onset and drives appetite in rodents and other mammals through hypothalamic activation of arcuate nucleus neurons (Abizaid and Horvath, 2012). Ghrelin’s action on feeding works through its activation on neuropeptide Y (NPY) and Agouti-related protein (AgRP) neurons and inhibitory effect on proopiomelanocortin (POMC) neurons (Cowley et al., 2003). The NPY/AgRP and POMC neurons ability to be modulated by opposing hormonal feedback mechanisms represents a fundamental neurocircuit to control appetite and satiety (Morton et al., 2014).

### Intergration of Signals Which Govern Feeding Behavior

In the brain, the hypothalamus is the critical modulator of homeostasis therefore controlling many vital functions such as feeding, reproduction or thermal regulation (Timper and Brüning, 2017). This is achieved via the integration of a wide range of endocrine, neural and metabolic signals, into effector responses of behavioral, autonomic, and endocrine nature. Other brain areas in the cerebral cortex and the brainstem (Schneeberger et al., 2014; Nectow et al., 2017) are also able to influence appetite and satiety, however, it is unclear if they achieve these functions independently of hypothalamic activity.

Rodent studies have evidenced that leptin action depends on specific receptors expressed in two distinct subsets of neurons in the arcuate nucleus (Cowley et al., 2001; Gropp et al., 2005; Luquet et al., 2005; Dietrich and Horvath, 2013). The orexigenic group of neurons (feeding-inducing) contains neuropeptides NPY and AgRP, with leptin suppressing their activity. The other group expresses anorexigenic peptides, cocaine and amphetamine related transcript (CART) and α-MSH (derived from POMC), with leptin inducing firing of these neurons and peptides release. Remarkably, AgRP and α-MSH are considered antagonistic ligands of a common receptor, the melanocortin 4 receptor (MC4R) (Dietrich and Horvath, 2013). MC4R is found exclusively in the brain (Balthasar et al., 2005). Activation of MC4R-expressing neurons decreases food intake, while their inhibition increases feeding and impairs leptin satiety responses in the brain.

In humans, functional magnetic resonance imaging studies have shown reduced hypothalamic responses upon glucose administration, and increased responses in limbic regions including the thalamus after overnight fasting (Smeets et al., 2005; Page et al., 2013).

## Olfaction as an Independent Influence in Driving Appetite, Response to Food Availabilty and Energy Balance

### Olfaction as an Important Driver of Appetite and Food-Seeking Behavior

Darwin provided evidence that a pleasurable or uncomfortable feeling can be detected in animals across a wide evolutionary spectrum, by observing their facial expressions. In his book, *The descent of man and selection in relation to sex* (Darwin, 1888), he says: “Those who believe in the principle of gradual evolution, will not readily admit that the sense of smell in its present state was originally acquired by man, as he now exists. He inherits the power in an enfeebled, and rudimentary condition, from some early progenitor, to whom it was highly serviceable, and by whom it was continually used. In those animals which have this sense highly developed, such as dogs and horses, the recollection of persons and of places is strongly associated with their odor, and we can thus perhaps understand how it is, as Dr. Maudsley has truly remarked, that the sense of smell in man is singularly effective in recalling vividly the ideas and images of forgotten scenes and places.”

There exits a tacit belief that the olfactory capability of humans does not match that of animals, which use smell as a warning and as a feeding signal. Current information does not support this contention (Mainland et al., 2014a, b) arguing that the human sense of smell is far more powerful than it was thought to be and that it plays an important regulatory in a wide range of human behaviors.

### Sense of Smell and Olfactory Processing as the Link Between Appetite, Food Reward and Metabolism

**FIGURE 3:**
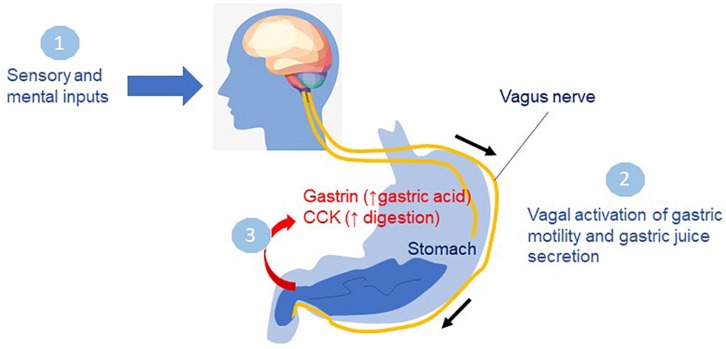
Sensory inputs on digestive processes. In the cephalic phase, smelling, seeing or thinking about food **(1)** drives the secretion of gastric juices in preparation for food intake via parasympathetic control through the vagus nerve **(2)**. In the gastric phase, food enters the stomach leading to secretion of gastrin, and other hormones promoting acid secretion in the stomach **(3)** preceding the intestinal phase when food leaves the stomach. Cholecystokinin (CCK) a major gastrointestinal hormone responsible for gallbladder contraction and pancreatic enzyme secretion functions in the small intestine to inhibit gastric emptying and allow digestion to occur.

The experience of food relies on the timely contribution of visual, auditory, olfactory, tactile and gustatory inputs. Food-related visual, olfactory and gustatory information converges on related areas of the brain, including the orbito-frontal cortex, insula and amygdala, where inputs from food experience influence behavior (reviewed in Riera and Dillin, 2016). Sensory inputs are well known to influence digestive processes (Figure 3). In the anticipatory or cephalic phase, sensory perception of food drives the secretion of gastric juices in preparation for food intake via parasympathetic control through the vagus nerve (Giduck et al., 1987).

Olfaction in particular, plays a dual role in food recovery and food perception in humans. In the “first contact” with food, odorants travel through the air to arouse olfactory sensory neurons in the nose via sniffing or orthonasal smelling, therefore leading to the localization and identification of food stimuli (Bojanowski and Hummel, 2012). In the “second contact” which occurs during food consumption, a large component of flavor perception is achieved through retronasal pathways: the olfactory molecules released during food breakdown through mastication enter the nasal cavity upon exhalation to stimulate olfactory sensory neurons (Bojanowski and Hummel, 2012).

It has been observed for several decades, that smell acuity varies depending on the feeding status. Hunger signals released during fasting are associated with increased olfactory perception, most likely to increase appetite for calorie dense foods (Albrecht et al., 2009; Tong et al., 2011; Cameron et al., 2012; Riera and Dillin, 2016). Food-related odors have also been shown to induce pre-oral salivation and cephalic responses such as gastric acid secretion and insulin release (Yeomans, 2006). While a meal is being consumed, a process termed sensory specific satiety is initiated and refers to the reduced pleasantness of the consumed food compared to new foods (Rolls et al., 1981; Critchley and Rolls, 1996). This variation in hedonic value may contribute to satiety and the decision to terminate the meal, together with post-ingestive signals released by the gastro-intestinal tract to the brain. Importantly, primate orbitofrontal cortex neurons stop responding to olfactory and gustatory cues after feeding to satiation (Critchley and Rolls, 1996), suggesting that satiety signals dampen olfactory neuronal activity.

As mentioned above, the hormone leptin, secreted by adipose tissue, suppresses appetite by inhibiting AgRP/NPY neurons activity in the hypothalamus. In mice lacking leptin (*ob/ob*) or leptin receptors (*db/db*), a voracious appetite elicited by high AgRP/NPY activity is associated by marked obesity and type 2 diabetes. Getchell et al. (2006) reported that in addition to a strong metabolic impairment, these mice presented a differential olfactory response to food ingestion. Interestingly, *db/db* and *ob/ob* mean time to smell and find food was approximately ten times shorter than wild-type. Remarkably, daily leptin injections are sufficient to suppress this enhanced olfactory phenotype. These data support a role for leptin in the regulation of olfactory-mediated pre-ingestive behavior by controlling olfactory sensitivity through a leptin-receptor (LepR) based mechanism.

Opposing leptin and its satiety action, the gastric hormone ghrelin is released in the stomach to stimulate appetite and food ingestion through its positive action on NPY/AgRP neurons and inhibitory effect on POMC neurons (Cowley et al., 2003). Ghrelin acts on olfactory processing centers in the brain of rats and humans by boosting olfactory acuity, most likely through direct binding of ghrelin receptors (GHSR) in these brain regions (Tong et al., 2011). Stereotaxic intracranial delivery of ghrelin improves olfactory sensitivity in rats and increased sniffing frequency (Tong et al., 2011). Ghrelin receptors have been localized in the main olfactory bulb and olfactory processing centers such as the amygdala and piriform cortex, both regions being important relays for olfactory information from the olfactory bulb (Becskei et al., 2007). How these neurons communicate with the hypothalamus remains to be determined. A potential excitatory circuit could mediate the enhanced olfactory acuity observed during fasting, and dependent upon ghrelin gastric secretion and action in the hypothalamus. In this scenario, ghrelin and leptin would promote opposite effects on olfactory sensing in order to adjust food scavenging behavior to metabolic states.

### Neuronal Control of the Smell Response to Food Availability

From the foregoing, it is clear that anticipatory signals provide rapid information about the availability and hedonic properties of food. To survive in a steady-state of access to food, two neuronal cell types play a key role in controlling feeding: NPY/AgRP and POMC-expressing cells residing in the arcuate nucleus of the hypothalamus. The elegant studies of Knight and coworkers (Chen et al., 2015) have shown that chemosensory detection of food resets the electrical state of these neurons within a very short time-frame supporting the notion that such behavior is under neuronal rather than humoral control. Using recent technological advances, Knight and colleagues performed awake-free behaving recording of hypothalamic neurons using an optical method called fiber photometry (Chen et al., 2015). Remarkably, it is the hedonic property and the energy content of the food, rather than its availability, that is sensed and processed, likely based upon smell and other sensory cues retained in memory.

Whereas AgRP and POMC neurons have hitherto been considered to be sensors of the nutritional state of the organism, activated by energy deficit to restore energy balance, the studies by Knight and coworkers show this not to be the case. They have clearly shown that resetting of the function of these neurons occurs well in advance of food consumption, by the detection of food alone. That sense of smell plays a key role in this regard, was demonstrated in fasted mice which were presented with food (peanut butter) in a closed container which avoided viewing by the animal. Rapid modulation of AgRP and POMC neuron discharges occurred within 1 min, mimicking the response to food presentation. If acess to the food was prevented following this response, the neural activity returned to baseline within 8 min. Thus sensory cues elicited by smell can modulate these neurons in anticipation of access to food, even if such access is not fulfilled. This insight indicates that foraging, the motivational search for food, is an instinct of the highest order, which elicits an immediate response rather than being a downstream event consequent upon food ingestion.

Do AgRP neurons drive food consumption via a smell-memory process? Does activation provide a stimulus analogous to learned hunger pangs? If so, this could drive food-seeking behavior as a means to avoid such sensations, leading to a repetitive cycle of appetitive behavior and consumption, with the hedonic properties of the food determining the magnitude of the response. The fast regulation of foraging described above is superimposed upon slow regulation controlled by nutrients, hormones and feeding/satiety cycles. This latter set of events is not the subject of the present paper.

### Independent Influence of Smell on Food-Seeking Behavior and Energy Balance

There is evidence for an independent influence of smell on food-seeking behavior in humans. One example is the suppression of appetite induced by smelling dark chocolate, an effect which correlates with changes in ghrelin levels in young females (Massolt et al., 2010). Another is the response to appetitive conditioning (exposure to a pleasant odor like vanilla) and aversive conditioning (exposure to an unpleasant odor like fermented yeast). This has been well studied with quantitative tools such as electromyography, electroencephalography, heart rate variation, skin conductance and functional magnetic resonance imaging (De Silva et al., 2012). Although the aversive response has been relatively easy to recognize (including typical facial expressions), the appetitive response, through which rewards and motivation are learned, has received less attention. Thus, whereas exposure of humans to an unpleasant odor elicits a measurable response, a pleasant odor fails to do so. Whether metabolic signals, such as fasting, can suppress neural responses to visual cues, or whether they affect the response to odors, is not known.

An important question remains. When considering olfaction and energy balance, can changes in olfactory sensitivity directly influence weight gain by manipulating neurocircuits converging to the hypothalamus? This question has been partially addressed in a mouse study. Riera et al. (2017) engineered mice harboring either reduced olfactory perception (hyposmia) or increased smell sensitivity (hyperosmia). Hyposmic mice were resistant to diet-induced obesity during high fat diet feeding. Chronic loss of smell perception in adult mice did not impact food intake but influenced satiety signals toward increased autonomic tone and higher fat burning mechanisms. Remarkably, in mice engineered to present increased olfactory sensing, weight gain upon normal chow was observed without altering food intake, leading over time to insulin resistance (Riera et al., 2017). These results suggest that olfactory circuits communicate with central neurons to regulate energy balance, and the precise nature of these circuits remains to be determined.

## Consequences of Loss or Abnormality of Sense of Smell

### Impact of the Loss of Sense of Smell

It is common experience that loss of smell (like that which occurs with a common cold) leads to a diminution of food enjoyment largely due to a loss of the tastefulness of food. Patients with smell disorders often report “eating disorders.” Studies which have examined food enjoyment before and after the onset of such disorders, have recorded appreciable lessening of enjoyment of food, associated with loss of appetite in many cases (Croy et al., 2014). Compensation for this is manifested by over-salting, over-sweetening or over-spicing of the food. A review compiles findings from a total of 14 eating disorders human studies, and demonstrate that firm conclusions on the association between loss of smell and eating disorders are difficult to reach (Islam et al., 2015) Partial loss of smell (hyposmia) or full loss of smell (anosmia) are found to be associated with anorexia nervosa, but not bulimia nervosa. However, these associations may be driven by the greater number of studies on anorexia compared to bulimia. Loss of smell in disease has been reported abundantly, with conflicting reports. It is important to distinguish if loss of smell is created by an acute insult (common cold, head injury, exposure to certain chemicals such as pesticides or chemotherapy) or developing progressively with another disturbance (chronic diseases or aging). Acute loss of smell in otherwise healthy humans is a documented cause of weight loss, anorexia nervosa, and depression (Henkin and Smith, 1971).

However, progressive loss of smell is associated with chronic obesity and inflammatory diseases such as Parkinson’s disease, Alzheimer’s disease, multiple sclerosis, hypertension, and natural aging (Graves et al., 1999; Ross et al., 2008; Wilson et al., 2009; Pinto et al., 2014). This suggests a strong correlation between early signs of neurodegeneration and olfactory decline (Riera and Dillin, 2016). Olfaction has been viewed as the “miner’s canary” of human brain health (Riera and Dillin, 2016), most likely because of its early failure due to high demand for neuronal regeneration in the olfactory epithelium and the olfactory bulb. To maintain olfactory function, stem cells pools constantly need to replace mature olfactory neurons in the nose where the half-life of these neurons is rather short (Pignatelli and Belluzzi, 2010). Therefore, olfactory loss may serve more generally as an indicator of deterioration in age-related regenerative capacity or as a marker of physiologic repair function.

Abnormal smell (parosmia), unpleasant smell (cacosmia) and phantom smells (phantosmia) are disturbances of olfaction which can lead to weight loss even though the relationship between weight loss and smell may be driven by psychological factors such as depression (Henkin and Smith, 1971; Palouzier-Paulignan et al., 2012; Oral et al., 2013). Of interest is the fact that congenital loss of sense of smell does not appear to affect food preferences. Loss of smell leads to loss of recognition of hazards such as smoke detection, difficulties with cooking, or accidentally eating rotten or spoiled food (Boesveldt et al., 2017). In a meta-analysis of studies on subjects with disorders of smell, the single-most prominent symptom in daily life, was a decrease in the enjoyment of food (Croy et al., 2014). In the Dutch Anosmia Association, about 40% of the members reported reduced appetite, but whether this condition can lead to alternative eating patterns is overall unclear (Toussaint et al., 2015). In certain cases, a shift toward healthier food options is reported among individuals which lost interest in food palatability (Aschenbrenner et al., 2008).

Here it is worth reflecting on the management of patients who are fed through a percutaneous endoscopic gastrotomy or a nasogastric tube, where there is no prior contact with the food (which, anyway, comes in a form which would not stimulate the senses), and hence there is no appetite response. Digestion and absorption would not proceed as would occur normally, but satiety does occur. Such patients are described as “forgetting their stomachs” (Pavlov and Thompson, 1982). To promote and improve digestion, Pavlov recommended restoring appetite, since “no other excitant can compare with the passionate craving for food.”

## Conclusion and Directions for Future Exploration

The importance of the contribution of Pavlov in exposing the “psychic” phase of digestion, which initiates all of the molecular events described above, cannot be overestimated (Pavlov and Thompson, 1902). This phase serves as being anticipatory of a reward and involves sensory triggers such as sight and smell, for which memory of prior experience bolsters the drive to seek food, the ingestion of which amplifies the motivating power of the preparatory phase.

It is surprising that Pavlov did not home in on smell as a major component of the “psychic’ phase of feeding. Other than remarking that the hands of those who fed his experimental animals should be free of the smell of flesh, he did not seek to isolate this important sensory dimension for experimentation. He could have blindfolded his dogs or he could have presented foods in a form which was unidentifiable other than the odor which it emitted, to isolate smell as a stimulus. Although Pavlov chose not to focus on specific sensory dimensions and assumed that neural afferent traffic accounted for all that he had observed, he was working on the cusp of an era of hormonal discoveries which soon would serve to embellish and clarify aspects that remained as open questions to him.

The foundations of the physiology of appetite laid down by Pavlov, have endured unchanged over the past century in the face of a vast amount of biological research. Appetite now involves integration of gastrointestinal physiology, neuroscience and cognitive and behavioral sciences. We propose that the multiple roles of sense of smell in the homeostatic and hedonic aspects of feeding have clearly entered center stage.

## Author Contributions

LF wrote the historical perspective. CR wrote the current knowledge on olfaction component of the hypothesis.

## Conflict of Interest Statement

The authors declare that the research was conducted in the absence of any commercial or financial relationships that could be construed as a potential conflict of interest.
